# Development and Validation of Two Cell-Based Reporter-Gene Assays for Determining the Bioactivity of Recombinant Human Thyroid-Stimulating Hormone Pharmaceutical Products

**DOI:** 10.3390/molecules30051037

**Published:** 2025-02-24

**Authors:** Lyuyin Wang, Jing Gao, Kaixin Xu, Jing Li, Chenggang Liang

**Affiliations:** 1 National Institutes for Food and Drug Control, No. 31, Huatuo Road, DaXing District, Beijing 102629, China; wanglvyin@nifdc.org.cn; 2 School of Life Science and Technology, China Pharmaceutical University, No. 639, Longmian Road, JiangNing District, Nanjing 210009, China; 17861405031@163.com (J.G.);

**Keywords:** reporter-gene assay, bioactivity, quality control, thyroid-stimulating hormone, method validation

## Abstract

To develop a cell-based in vitro thyroid-stimulating hormone (TSH) biological activity assay that can simulate in vivo pharmacodynamic mechanisms, we constructed two HEK293-TSHR cell lines based on two main cell signaling pathways (Gαs-cAMP-PKA and Gαq/11-PLC-Ca^2+^) that TSH depends on for its in vivo physiological function. These cell lines stably expressed the luciferase reporter driven by the cAMP response element (CRE) and nuclear factor of activated T cells (NFAT) response element, and two reporter-gene assays (RGAs) were correspondingly established and validated. The two transgenic genes could measure signals produced from the simulation of the in vivo effects of TSH from the Gαs-cAMP and Gαq/11-PLC pathways after TSH activation. TSH showed a good dose–response relationship in these two cell lines and conformed to the four-parameter model. We optimized the critical experimental parameters of these two methods and performed comprehensive methodological validation according to the International Council for Harmonization (ICH) Q2 (R1) guidelines, the Chinese Pharmacopoeia, and the United States Pharmacopoeia. The two methods showed good specificity, accuracy, precision, and linearity and can be used to aid in assessments of the biological activity of TSH drugs, product characterization, final product release, stability studies, and comparability studies for biosimilar applications.

## 1. Introduction

Thyroid-stimulating hormone (TSH) is a glycoprotein hormone secreted by the anterior pituitary [1] and is an important endocrine hormone for maintaining the normal physiological function of the thyroid. TSH can stimulate the synthesis and secretion of thyroid hormones, induce iodine uptake in the thyroid, and promote thyroid growth and development [2,3], and it is physiologically important in regulating basal metabolism in the body. The release of TSH is regulated by the classic hypothalamic–pituitary–thyroid axis, which is vital in maintaining the homeostasis of metabolic functions within the body [4]. The hypothalamic thyrotropin-releasing hormone (TRH) causes TSH to be released from the pituitary, and the resulting increased levels of TSH stimulate the thyroid follicular cells to produce more thyroid hormone in the form of triiodothyronine (T3) and thyroxine (T4). This, in turn, inhibits the production of TSH [5]. Recombinant human TSH (rhTSH) is a recombinant protein obtained via genetic recombination and in vitro expression and has mechanisms of action and biological characteristics similar to endogenous human TSH. In clinical practice, rhTSH is mainly used for adjuvant treatment and follow-up diagnosis of differentiated thyroid carcinoma (DTC) patients without distant metastasis after postoperative iodine-131 ablation of residual thyroid tissue [6,7]. Compared with traditional thyroid-hormone withdrawal methods, rhTSH injection can increase serum TSH levels more safely and rapidly and can minimize the suffering of patients caused by discontinuation of thyroid hormones [8,9]. Findings from the last 30 years of clinical application of rhTSH have resulted in it being included in many thyroid cancer-related clinical guideline recommendations around the world [10]. Thyroid cancer is the most common malignant tumor [11]; DTC accounts for more than 95% of all thyroid cancers [12]. Currently, only one rhTSH drug (Thyrogen, Genzyme, Inc., Cambridge, MA, USA) is available globally, one which was approved for marketing in the U.S.A. in 1998 [13]. Many rhTSH drugs are currently in different clinical trial stages [14,15].

Biological activity can reflect the accuracy of the higher order structure of rhTSH molecules, the integrity of biological function, and the inter-batch consistency of products [16], and it is an effective and essential method for assessing the quality of rhTSH products. Hence, developing a rapid, simple, sensitive, accurate, and stable in vitro biological activity assay is essential. The early TSH activity assay measured the increase in iodine-131 in mouse blood after dosing [17]. Because animal experiments involve long cycles, sizeable inter-individual variability, cumbersome operations, and high variability, and, additionally, do not conform to 3R principles [18], a consensus has been reached to develop alternatives to animal experiments. In 2022, Liu et al. developed a luminescence resonance energy transfer (LRET)-based TSH activity assay [19]. Although this assay is simple and has high specificity and sensitivity, it is still a content measurement assay and cannot simulate in vivo pharmacodynamics and thus truly reflect the biological activity of TSH. The use of cell-based in vitro assays to replace animal experiments is a trend in the development of quality control of biologics in China and other countries. Most reported in vitro cellular assays use TSH to stimulate the cAMP pathway and use ELISA to measure the TSH-induced intracellular cAMP increase to evaluate TSH biological activity. Although this is based on TSH’s in vivo mechanisms of action (MOA), the operation is cumbersome, variability is high, and sensitivity is low. A cell-based reporter-gene assay uses drug effector signaling pathways. This not only examines live cell function but also is closer to the physiological state, and it intuitively and directly reflects the targeting effects of the drug. This method has high sensitivity, stability, and accuracy, and it has been widely used to measure the in vitro biological activity of therapeutic protein drugs in recent years [20,21].

TSHR belongs to the G-protein coupled 7 transmembrane leucine-rich repeat (LGR) subfamily and is the first critical substrate in the TSH signaling pathway [22]. Moreover, TSHR is one of the key targets for the correction of diseases caused by aberrant TSHR function (such as Grave’s hyperthyroidism, non-autoimmune hyperthyroidism, and thyroid cancer) [23]. It is well established that TSH can activate four G protein subtypes (Gαs, Gαq/11, Gβγ, and Gα12/13) that are coupled to the TSH receptor (TSHR) [24]. Among them, the most canonical signaling pathway is the Gαs-mediated cAMP-PKA pathway [3]. Gαs activation causes the intracellular second messenger cAMP concentration to increase, followed by PKA activation and phosphorylation of cAMP response element binding protein (CREB) and other transcription factors [25]. Phosphorylated CREB recognizes the specific response element CRE on gene promoters to regulate the transcription of downstream significant genes that participate in thyroid-hormone synthesis, cell growth, and differentiation, including sodium iodide symporter (NIS), thyroglobulin (TG), and thyroid peroxidase (TPO). Gαq/11 activation causes phospholipase C (PLC) activation, which enhances the production of the second messenger inositol 1,4,5-trisphosphate (IP3) [26]. Then, this triggers intracellular calcium-ion release and rapid translocation of the transcription factor NFAT into the cell nucleus and binding to a specific response element NFAT-RE on gene promoters to drive the expression of downstream genes. At the same time, protein kinase C (PKC) and its downstream effector gene MAPK are also activated, which activates dual oxidase 2 (DUOX-2), produces hydrogen peroxide, and promotes TG iodination to achieve regulation of critical steps in thyroid hormone synthesis. Hence, this is another essential signaling pathway of TSH activation, the PLC-Ca^2+^ pathway. TSH regulates the transcription and expression of related enzymes and precursor genes through these two signaling pathways [27] to achieve its main biological functions—regulation of iodine uptake, thyroid-hormone synthesis, and targeting of cell growth and differentiation [28,29].

Based on our understanding of these two signal transduction pathways (cAMP-PKA and PLC-Ca^2+^), which are intimately associated with the biological function of TSH, we selected CRE and NFAT as response elements to construct reporter-gene vectors, which were separately transfected into HEK293 cells together with the TSHR expression vector. Two reporter-gene cell lines were thus obtained, which were the HEK293-TSHR/CRE-Luc cells, which stably express human TSHR and CRE-regulated luciferase, and HEK293-TSHR/NFAT-Luc, which stably expresses human TSHR and NFAT-regulated luciferase. After rhTSH binds to TSHR on the cell membranes of the two aforementioned HEK293 cells, it increases cAMP and Ca^2+^ concentrations, which promotes the expression of CRE and the NFAT-driven luciferase reporter gene. Measuring the intensity of fluorescence signals can thus characterize the biological activity of TSH. We optimized the critical experimental parameters of these methods and performed comprehensive methodological validation according to ICH Q2 (R^2^) guidelines, the Chinese Pharmacopoeia, and the United States Pharmacopoeia. The results showed that these two methods are simple, have high sensitivity, good accuracy, and precision, and can be used for evaluation of in vitro pharmacodynamics and quality control of rhTSH products.

## 2. Results

### 2.1. Generation and Selection of the HEK293-TSHR/CRE-Luc and HEK293-TSHR/NFAT-Luc Cell Lines

**Figure 1:**
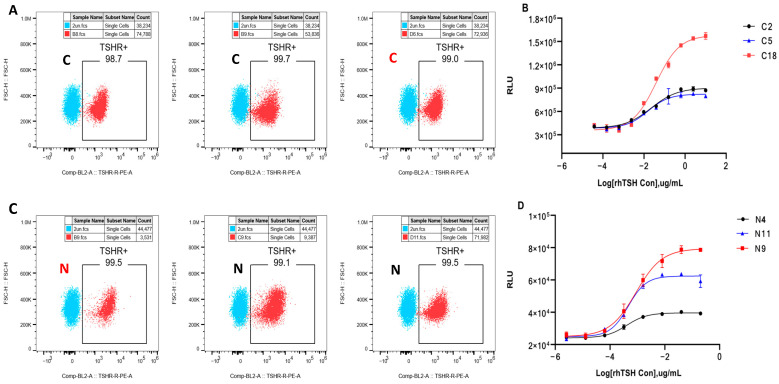
HEK293-TSHR/CRE-Luc and HEK293-TSHR/NFAT-Luc cells responsive to rhTSH. HEK293-TSHR/CRE-Luc and HEK293-TSHR/NFAT-Luc cell lines were analyzed by flow cytometry for the expression of hTSH receptor on the cell surface ((**A**) and (**C**), respectively). Three HEK293-TSHR/CRE-Luc monoclones (C2, C5, and C18) and three HEK293-TSHR/NFAT-Luc monoclones (N4, N11, and N9) were stimulated with serially diluted rhTSH ((**B**) and (**D**), respectively), and the dose–response curves are shown.

After HEK293 cells were infected by packaged viruses, three HEK293-TSHR/CRE-Luc monoclonal cells (C2, C5, and C18) and three HEK293-TSHR/NFAT-Luc monoclonal cells (N9, N4, and N11) were obtained via drug selection and limiting dilution. As shown in Figure 1A, flow cytometry showed that TSHR expression on the surfaces of the three HEK293-TSHR/CRE-Luc cell lines was more than 97%, of which the C18 monoclone had the highest TSHR expression (98.8%). Figure 1C shows that among the three HEK293-TSHR/NFAT-Luc monoclonal cells, the N9 monoclone had the highest TSHR expression (99.4%). As shown in Figure 1B,D, the six monoclonal lines all responded to rhTSH stimulation, among which the C18 and N9 monoclonal lines presented greater responsiveness and a higher S/N ratio. Therefore, we used the C18 HEK293-TSHR/CRE-Luc monoclonal cells and N9 HEK293-TSHR/NFAT-Luc monoclonal cells for subsequent method optimization and validation.

### 2.2. Optimization of the Two Reporter-Gene Assays

#### 2.2.1. CRE Assay

**Figure 2:**
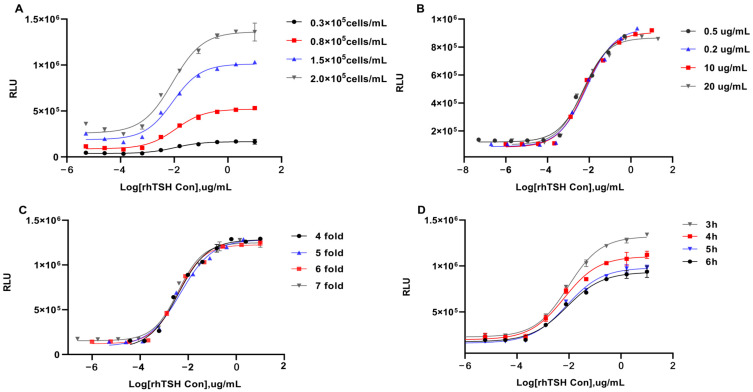
Optimization of the RGAs based on HEK293-TSHR/CRE-Luc cells. HEK293-TSHR/CRE-Luc cells were adjusted to 0.3, 0.8, 1.5, and 2.0 × 10^4^ cells per well and incubated with serial dilutions of rhTSH. The dose–response curves were recorded to determine the optimal cell density (**A**). Cells were treated with gradient dilution of rhTSH with different initial concentrations (0.5, 0.2, 10, and 20 μg/mL) (**B**). After fixing the initial concentration at 10 μg/mL, rhTSH was diluted in 4-, 5-, 6-, and 7-fold serial dilutions to 10 concentration levels (**C**). RLU was measured after the test plates were incubated for different times (3, 4, 5, and 6 h) (**D**).

Cells were seeded on a plate at densities of 0.3 × 10^5^, 0.8 × 10^5^, 1.5 × 10^5^, and 2.0 × 10^5^ cells/mL and used to measure the biological activity of the same rhTSH sample. As shown in Figure 2A, when the cell density was 0.8 × 10^5^ cells/mL, the R^2^ was 0.991, the SNR was the highest at 11.0, and EC_50_ was the lowest. The rhTSH was used to stimulate HEK293-TSHR/CRE-Luc cells at starting concentrations of 0.5, 2, 10, and 20 μg/mL. As shown in Figure 2B, when the starting concentration was 10 μg/mL, the dose–response curve appeared as a typical S shape, the points on the curve were uniformly distributed, the SNR was the highest, and R^2^ was 0.992. Therefore, the optimal starting concentration was determined to be 10 μg/mL. Dilution factor optimization results (Figure 2C) showed that a typical S curve could only be obtained with cells stimulated with six-fold serially diluted rhTSH, and curves obtained using the other three dilution factors did not show a significant upper plateau or lower plateau. Drug stimulation time optimization results (Figure 2D) showed that the S curve could be obtained by rhTSH stimulation of cells for 3, 4, 5, and 6 h. At 4 h, EC_50_ was the lowest, and the SNR was the highest. Therefore, 4 h was selected as the optimal drug stimulation time.

#### 2.2.2. NFAT Assay

**Figure 3:**
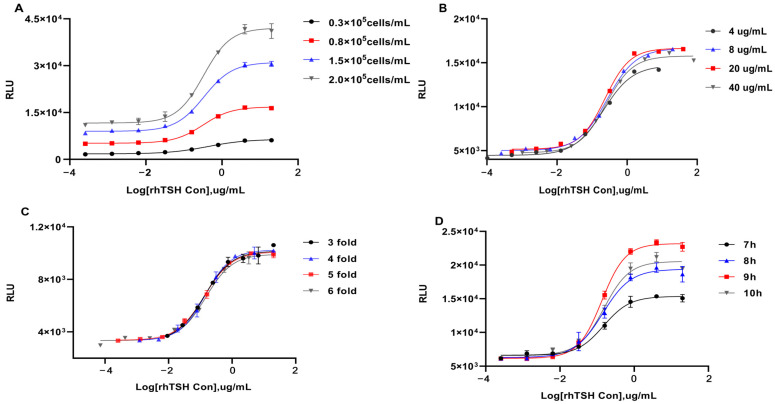
Optimization of the RGAs based on HEK293-TSHR/NFAT-Luc cells. The full dose–response curves are shown with the cells seeded at 8000 per well with various initial concentrations as indicated (**A**). RhTSH was diluted in 3-, 4-, 5-, or 6-fold serial dilutions to generate 10 concentration points, with an initial working concentration of 20 μg/mL. The dose–response curve is shown in (**B**). The dose–response curve shown was measured in the presence of different cell numbers as indicated (**C**) and at incubation times of 7, 8, 9, and 10 h shown in (**D**).

Similarly, cells were seeded on a plate at densities of 0.3 × 10^5^, 0.8 × 10^5^, 1.5 × 10^5^, and 2.0 × 10^5^ cells/mL, and the NFAT assay was used to measure the biological activity of the same rhTSH sample. As shown in Figure 3A, the typical S curve could be obtained from four cell densities, with an SNR of 3.5. When the cell density was 0.8 × 10^5^ cells/mL, the R^2^ was the highest at 0.995, and the EC_50_ was the lowest at 0.32. The rhTSH was used to stimulate HEK293-TSHR/NFAT-Luc cells at starting concentrations of 4, 8, 20, and 40 μg/mL. As shown in Figure 3B, when the starting rhTSH concentration was 20 μg/mL, the dose–response curve appeared as a typical S shape, R^2^ was 0.995, and the points on the curve were uniformly distributed. Therefore, the optimal starting concentration was determined to be 20 μg/mL. Figure 3C shows the dilution factor optimization results. A typical S curve with complete upper and lower plateaus could only be obtained with HEK293-TSHR/NFAT-Luc cells stimulated with five-fold serially diluted rhTSH. Therefore, five-fold was selected as the optimal serial dilution factor. The stimulation time optimization results (Figure 3D) showed that the SNR of the curve was at its highest at 3.78 when the stimulation time was 9 h. Therefore, the optimal incubation duration for rhTSH stimulation of HEK293-TSHR/NFAT-Luc cells was determined to be 9 h.

### 2.3. Methodological Validation

#### 2.3.1. Specificity

**Figure 4:**
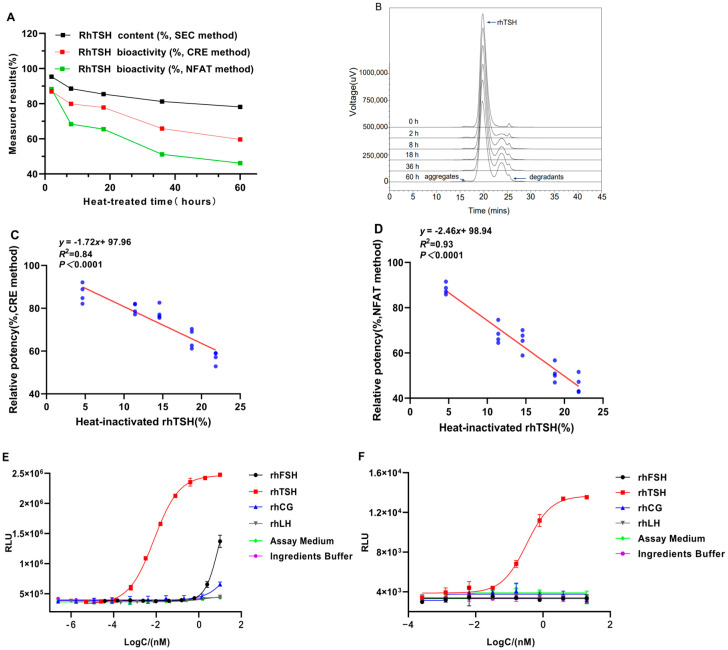
Specificity analysis of two new RGAs. (**A**) Relative potency estimated for CRE RGA and NFAT RGA, and the content measured via size-exclusion high performance liquid chromatography (SE–HPLC) of rhTSH samples kept at 60 °C for different durations of time (h). (**B**) Analysis of aggregates, degradants, and rhTSH contents in heat-inactivated samples via SEC–HPLC. Correlation analysis of the heat-inactivated rhTSH content with biological activity obtained using the CRE (**C**) and NFAT assays (**D**). Dose–response curves of the ingredients buffer, assay medium, rhFSH, rhTSH, rhCG, and rhLH, as determined with CRE (**E**) and NFAT RGAs (**F**).

After rhTSH was incubated at 60 °C for 0, 2, 8, 18, 36, and 60 h, the two reporter-gene assays were used to measure its biological activity. As shown in Figure 4A, when the CRE assay was used, compared with the untreated reference standard, the relative potencies of the treated reference standard decreased with increasing treatment duration, and they were 86.97, 79.91, 77.86, 65.81, and 56.99% after treatment at 60 °C for 2, 8, 18, 36, and 60 h, respectively. The relative potencies of the heat-degraded reference standard measured using HEK293-TSHR/NFAT-Luc cells were 88.31, 68.39, 65.49, 51.15, and 46.19% after treatment at 60 °C for 2, 8, 18, 36, and 60 h, respectively. The rhTSH contents measured using size exclusion chromatography were 95.36, 88.58, 85.43, 81.24, and 78.16%, respectively. Thus, the rhTSH main component content gradually decreased with increasing heat duration, and the contents of dissociated subunits and aggregates gradually increased, which was consistent with the biological activity variation trend obtained using the two RGAs that showed a positive correlation. In comparing the results of the three methods on the same heated sample, after heating for 8 h, the biological activity measured using the main component content was higher than found with the CRE assay. The biological activity measured using the CRE assay at different time points was stably higher by 12% than that associated with the NFAT assay. To further analyze the cause of the aforementioned experimental phenomenon, we used the heat-inactivated rhTSH content% as the *x*-axis coordinate and the biological activity% obtained from four repeated measurements using the two RGAs as the *y*-axis coordinate for correlation analysis. The results are shown in Figure 4C,D, and the P values are all less than 0.001. The biological activity% measured using the two RGAs was correlated to the heat-inactivated rhTSH content%, and the correlation between the NFAT measurement and heat-inactivated rhTSH% was higher than the CRE assay.

The rhFSH, rhLH, rhCG, and rhTSH preparations, the buffer solution, and the measurement culture medium were used to stimulate the HEK293-TSHR/CRE-Luc cells. As shown in Figure 4E, there was no response for the rhLH or rhTSH excipient blank (ingredients buffer) or the measurement culture medium, and only rhFSH and rHCG showed a response for this assay. However, the response values did not result in an upper plateau. The HEK293-TSHR/NFAT-Luc cell-based RGA was used, and the rhFSH, rhLH, rhCG, and rhTSH preparations, the buffer solution, and the measurement culture medium showed no responses (Figure 4F). Of the two RGAs, NFAT had better specificity.

#### 2.3.2. Accuracy

The newly constructed CRE and NFAT RGAs were validated. Two testers separately measured the relative potency of test solutions of five different potency levels (50, 80, 100, 125, and 200%), and the results all passed the reliability test. As shown in Table 1 and Table 2, the relative bias of relative potency measured within the 50–200% potency range of the two assays was −8.2% to −3.2% (CRE assay) and −2.7 to 6.2% (NFAT assay). This shows that the relative potency measurements were close to the theoretical values and that the two methods have good accuracy.

#### 2.3.3. Precision

Two testers measured the relative potency of the five aforementioned test solutions within the same day for 2 days. Each tester had to prepare two sets of test solutions with the same potency level on the same day, and two cell passages were used. Each cell passage was used for two 96-well plate tests, and the geometric mean was calculated. The logarithmic values of the various potency level results were used to calculate the GCV (%), and the Chi-square test was used to calculate the upper limit of the 95% confidence interval of GCV. As shown in Table 3 and Table 4, the GCV of relative potencies for each potency level in the eight experiments was 4.2 to 9.8% (CRE assay) and 5.2 to 7.4% (NFAT assay), and the corresponding 95% confidence interval upper limits were less than 20 and 15%, respectively.

#### 2.3.4. Linearity and Range

**Figure 5:**
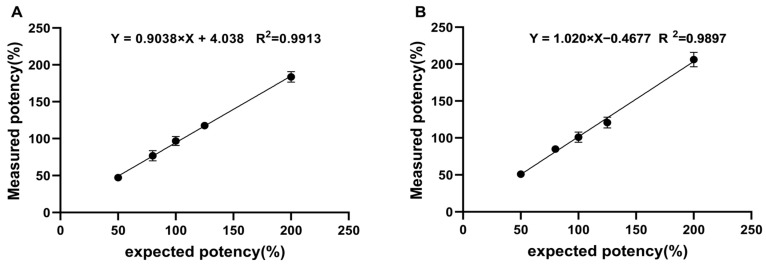
Linearity of the two RGAs. In the CRE (**A**) and NFAT methods (**B**), five samples at different relative bioactivity levels were analyzed, and the measured and expected potency of rhTSH showed a strong linear correlation. Each point indicates the average of eight independent replicates.

Linear regression was carried out on the natural logarithmic values of the theoretical values of the five potency levels and the natural logarithmic values of their corresponding potency measurements. The results are shown in Figure 5. The linear regression formulas of the two assays are Y = 0.904X + 4.038 (R^2^ = 0.9913) and Y = 1.020X − 0.468 (R^2^ = 0.9897). The slopes were close to 1.0, and R^2^ was greater than 0.98. This shows that the two assays have good linearity within the 50–200% potency level.

### 2.4. Application of the Reporter-Gene Assays

The two newly constructed RGAs were used to measure the biological activity of three batches of rhTSH stock solutions and three batches of rhTSH injections. Each batch was measured three times in parallel. All test results obtained using the two assays passed the reliability test. The relative standard deviation of the three repeated relative potency measurements of three batches of stock solutions (DS1, DS2, and DS3) and three batches of injections (DP1, DP2, and DP3) were all less than 10% (Table 5), showing that these two methods had good repeatability in measuring the in vitro biological activity of rhTSH and can be used for the relative biological activity measurement of rhTSH stock solutions and product samples.

## 3. Discussion

In this paper, Gαs-cAMP-PKA and Gαq/11-PLC-Ca^2+^, two signal transduction pathways that are intimately associated with TSH function, were successfully used to construct double transfected HEK293-TSHR stable lines containing CRE or NFAT response elements (RE) tagged to a modified form of the luciferase reporter, and two RGAs that are highly correlated with the in vivo MOA of TSH were established.

In the optimization of the two reporter-gene assays, four parameters, namely, cell density, the starting concentration of rhTSH, rhTSH dilution factor, and incubation time, were examined individually to determine the optimal RGA conditions. The experimental parameters of the CRE assay (and NFAT assay) were finally determined, with a starting working concentration of 10 μg/mL (20 μg/mL for NFAT assay) for rhTSH, 10 dilutions in a six-fold (five-fold for NFAT assay) series, and a drug action time of 4 h (9 h for NFAT assay). As shown in Figure 2 and Figure 3, the SNR values of the four-parameter curve obtained using the CRE and NFAT assays were 10 and 4, respectively. Hence, compared with the NFAT assay, the CRE assay produces signals faster with a higher signal intensity. As the drug concentration increases, the detection limit decreases, the detection range becomes broader, and the sensitivity and detection efficiency increase. These results were similar to CHO cell results in past studies [25]. Moreover, this shows that the PKA-Gαs pathway is the main pathway of TSH activation.

After using the analysis of variance and F test for reliability testing of the results of the analysis, we performed sufficient methodological validation of the two assays. To validate the specificity of the two RGAs, we analyzed the activity of the rhTSH reference standard after heating on different days. With increasing degrees of damage, the bioactivity determined by RGA and the main component content determined using size exclusion chromatography showed identical decreasing trends. After rhTSH was incubated at 60 °C for 8 h, the main component content and activity began to decrease considerably. For the same heat-inactivated sample, the biological activity measured using the NFAT assay and the main component content were lower than those measured using the CRE assay (Figure 4A). Hence, the two RGAs can not only distinguish between rhTSH and its heat byproducts, reflecting the correlation between the rhTSH structure and function, but they can more sensitively reflect sample stability than physicochemical methods. In addition, the NFAT assay can more sensitively reflect reduced activity due to heat destruction and has a higher detection capacity for heat-inactivated rhTSH. The correlation analysis results showed that the biological activity obtained using the two RGAs was correlated with the heat-inactivated rhTSH content, and the NFAT assay showed a stronger correlation. This shows that high temperature-induced rhTSH molecular structure changes have some inhibitory effects on the two signaling pathways and have a more significant effect on the Gq/11-mediated PLC-Ca^2+^ signaling pathway. The rhTSH consists of an α-subunit (92 amino acids) and a β-subunit (112 amino acids), bonded non-covalently. At high temperatures, the degradation mechanism of rhTSH is mainly dissociation, and the dissociated subunits are the main heat-destruction products. Studies have reported that free TSH α- and β-subunits can bind to TSHR, and free α-subunits will inhibit TSHR activity [30]. The inhibitory effects of free α subunits on TSHR may be one of the reasons why activation of the two aforementioned signaling pathways was inhibited.

Because TSH, LH, follicle-stimulating hormone (FSH), and HCG all belong to the glycoprotein family, they all contain a common α subunit and a hormone-specific β subunit. Their corresponding receptors, namely, FSHr, LHr, hCGr, and TSHr, are members of the rhodopsin-like G protein-coupled receptor (GPCR) family [31]. Homologous genes code the β subunits and corresponding receptors of the aforementioned glycoprotein hormones. Therefore, rhFSH, rhLH, and rhCG were selected to measure the specificity of the two assays. The results showed no rhFSH, rhLH, or rhCG response in the NFAT assay (Figure 4F). However, high concentrations of rhFSH and rhCG showed responses in the CRE assay, but a typical four-parameter curve could not be obtained (Figure 4E). On the one hand, this shows that the NFAT assay had better specificity. On the other hand, this shows that high concentrations of rhFSH and rhCG could bind to TSHR and activate the Gαs-coupled cAMP-PKA pathway. Our experimental results also showed that rhFSH has stronger activating effects on the cAMP-PKA pathway than does rhCG.

The two RGAs showed good dilution linearity within the relative potency range of 50–200%, with the relative bias not exceeding ±10%, and the relative accuracy was good. As shown in Table 3 and Table 4, the two methods did not have high variability, and the corresponding 95% confidence interval upper limits were less than 20% (CRE assay) and 15% (NFAT assay), respectively. The precision of the NFAT assay was slightly better than that of the CRE assay. The RSD of repeated bioactivity measurements of three batches of stock solutions and three batches of injections were all less than 10% (Table 5), indicating that both methods had good repeatability and could be used as routine methods to measure rhTSH biological activity.

## 4. Materials and Methods

### 4.1. Cell Lines and Reagents

CRE reporter HEK293 cell line expressing luciferase and NFAT reporter HEK293 cell line expressing luciferase were supplied by Mijia Co., Ltd. (Beijing, China). DMEM culture medium, FBS, phosphate buffered saline (PBS), and puromycin were purchased from Gibco, Inc. (Grand Island, NY, USA). Hygromycin B was purchased from Life Technologies Corporation (Shanghai, China). The international rhTSH standard (03/192) for bioassays was purchased from NIBSC. The Steady-Glo Luciferase Assay System 2520ES Kit was purchased from Promega, Inc. (Madison, WI, USA). The pLV[Exp]-Hygro-EF1A>hTSHR plasmid was purchased from Beijing Mijia Co., Ltd. The rhTSH products and other hormones, including follicle stimulating hormone (FSH), luteinizing hormone (LH), and human chorionic gonadotropin (HCG), were archived biopharmaceuticals that had been preserved at 4 °C or −80 °C in our laboratory.

### 4.2. Construction of HEK293-TSHR/CRE-Luc and HEK293-TSHR/NFAT-Luc Cell Lines

After pLV[Exp]-Hygro-EF1A>hTSHR plasmid was packaged into lentiviruses, they were used to infect the HEK293/CRE-Luc and HEK293/NFAT-Luc stable cell lines. First, 100 μg/mL hygromycin B was used to select the aforementioned cells until stably transfected cells were obtained. Limiting dilution was used to obtain monoclonal cell lines. The cell lines were named HEK293-TSHR/CRE-Luc and HEK293-TSHR/NFAT-Luc and kept in our laboratory. Finally, flow cytometry was used to analyze TSHR expression on cell surfaces and confirm the positive monoclonal cells.

### 4.3. Cell Culture

HEK293-TSHR/CRE-Luc and HEK293-TSHR/NFAT-Luc cell lines were cultured in DMEM medium supplemented with 10% FBS, 100 μg/mL Hygromycin B, and 1 μg/mL puromycin. To maintain good condition, both cell lines should be passed every 3 to 4 days, and the densities for passaging should be 200,000 cells/mL for HEK293-TSHR/CRE-Luc cells and 400,000 cells/mL for HEK293-TSHR/NFAT-Luc cells.

### 4.4. RGA Procedure

The reporter-gene assays based on the HEK293-TSHR/CRE-Luc cell line and the HEK293-TSHR/NFAT-Luc cell line were named the CRE and NFAT assays, respectively. The cells were resuspended in DMEM culture medium containing 10% FBS, 100 μg/mL hygromycin B, and 1 μg/mL puromycin to obtain a cell suspension with a density of 8 × 10^4^ cells/mL. The cells were seeded in a 96-well white cell culture plate at 100 μL/well and cultured for 16–24 h in a 37 °C, 5% CO_2_ cell culture incubator. Then, the plates were removed, and the culture medium was discarded. The measurement culture medium (DMEM culture medium containing 0.1% BSA) was used to dilute the rhTSH sample and reference standard (rhTSH international reference standard) to a starting concentration of 10 μg/mL (20 μg/mL for the NFAT assay), and 6-fold (5-fold for the NFAT assay) serial dilutions were made to obtain 10 (eight for the NFAT assay) dilutions. These dilutions were added at 100 μL/well into the seeded 96-well plates. The culture plates were incubated in a 37 °C, 5% CO_2_ cell culture incubator for 4 h (9 h for the NFAT assay) and then removed. Then, 100 μL of Steady-Glo Luciferase Assay reagent.was added to each well, and the plates were allowed to react in the dark for 15 min. The EnSpire microplate reader was used to measure relative luciferase units (RLU).

### 4.5. Data Analysis

SoftMax Pro software 7.1.2 was used to analyze the experimental data, and GraphPad Prism software 9.5 was used to plot the graph. The logarithm base 10 values of rhTSH concentration were used as the *x*-axis coordinate and RLU was used as the *y*-axis coordinate to plot a four-parameter dose–response curve. The relative bioactivity was expressed as the ratio of the concentration for 50% of the maximal effect (EC_50_) of the reference to that of the sample in the constraint model. The signal-to-noise ratio (SNR) was expressed as the ratio of the upper asymptote to the lower asymptote. Analysis of variance and an F test were used to test the reliability of the results. The determination criteria were the following: R^2^ of the fitted 4-parameter curve of ≥0.98, regression item should be highly significant (*p* < 0.01), and deviation from parallel should not be significant (*p* ≥ 0.01). The reliability test results can be used to calculate the percentage of relative biological activity.

### 4.6. Specificity

To validate that the aforementioned reporter-gene assays are specific for rhTSH, a sample specificity test was performed with the rhFSH, rhLH, rhCG, and rhTSH; the blank buffer solution; and the measurement culture medium.

In addition, the specificity test involves interference from possible degradation products as TSH undergoes structural changes under high-temperature treatment. To further validate that this method can reflect the relationship between the TSH structure and function, we aliquoted one batch of rhTSH stock solution into 1000 μL vials. We placed them severally in a 60 °C water bath for 2, 8, 18, 36, and 60 h. The two aforementioned reporter-gene assays were used to measure the relative potency. Size exclusion chromatography was used to measure the content of rhTSH after incubation at 60 °C for different times.

### 4.7. Accuracy, Precision, and Linearity

The rhTSH international reference standard was used to make a 10 μg (20 μg for the NFAT assay) per mL solution using the measurement culture medium as the method validation reference standard. The measurement culture medium was used to prepare a series of solutions with starting concentrations of 5, 8, 10, 12.5, and 20 μg/mL (10, 16, 20, 25, and 40 μg/mL for the NFAT assay), which were used as test solutions with five different potency levels (50, 80, 100, 125, and 200%). The five relative potency levels have uniform intervals on a logarithmic scale. Two testers used two cell passages to measure the relative potency of the five test solutions within a period of 2 days. Each potency level was measured eight times, and the geometric mean was calculated. To evaluate the accuracy we determined the relative bias via the following formula: RB% = (relative potency measurement/theoretical relative potency − 1) × 100%. For intermediate precision, the geometric coefficient of variation (GCV, %) of the various potency level results was used, and the Chi-square test was used to calculate the upper limit of the 95% confidence interval of the GCV. The logarithm of the relative potency values of the five test solutions was used as the *x*-axis coordinate, and the theoretical logarithmic value of relative potency was used as the *y*-axis coordinate for linear regression analysis.

## 5. Conclusions

In recent years, there has been rapid progress in small molecule or antibody agonists and antagonists that target TSHR [1,22,23,25]. These drugs can bind to TSHR and induce selective signal transduction to mediate related physiological effects [32]. The two transgenic cell lines constructed in this study can activate the Gαs and Gαq/11 pathways of TSHR. This provides a tool for screening and MOA studies on the aforementioned small molecules or antagonists and agonists, as well as technical tools for developing novel antibodies or small molecule drugs that target autoimmune diseases caused by abnormal TSHR function.

TSH shows efficacy in osteoporosis, thyroid cancer, polycystic ovary syndrome, and other diseases [33,34]. In the future, modifying the TSH molecular function or developing safe and effective TSH analogs will have high diagnosis and treatment value in clinical practice. The RGAs constructed in this study are simple and efficient, have high precision and accuracy, and have potential applications in the production and R&D of rhTSH drugs, process control, stability studies, finished product releases, and comparability studies for biosimilar applications. Moreover, they could be used as references for biological activity measurements of rhTSH products.

## Figures and Tables

**Table 1:** Validation of the accuracy for detecting the relative potency of rhTSH using the CRE assay.

Potency Level	Run	Relative Potency			Relative Bias		
		Mean	Lower Confidence	Upper Confidence	Mean	Lower Confidence	Upper Confidence
50%	8	47.1%	49.8%	44.3%	−5.8%	−11.4%	−0.7%
80%	8	76.7%	81.6%	71.9%	−4.1%	−10.3%	1.6%
100%	8	96.8%	101.1%	92.5%	−3.2%	−7.6%	0.6%
125%	8	117.6%	120.1%	115.0%	−6.0%	−8.1%	−4.0%
200%	8	183.7%	188.7%	178.7%	−8.2%	−10.7%	−5.7%

**Table 2:** Validation of the accuracy of detecting the relative potency of rhTSH using the NFAT assay.

Potency Level	Run	Relative Potency			Relative Bias		
		Mean	Lower Confidence	Upper Confidence	Mean	Lower Confidence	Upper Confidence
50%	8	51.0%	53.1%	48.9%	2.0%	−2.6%	6.3%
80%	8	85.0%	87.8%	82.1%	6.2%	2.5%	9.7%
100%	8	101.0%	105.9%	96.1%	1.0%	−3.9%	5.7%
125%	8	121.7%	126.6%	116.7%	−2.7%	−6.6%	1.1%
200%	8	208.4%	216.8%	200.0%	4.2%	−0.2%	8.3%

**Table 3:** Intermediate precision validation of the CRE assay.

Potency Level	Run	GSD	CIGSD	GCV	CIGCV
50%	8	1.0890	1.1654	8.9%	16.5%
80%	8	1.0980	1.1828	9.8%	18.3%
100%	8	1.0657	1.1211	6.6%	12.1%
125%	8	1.0332	1.0604	3.3%	6.0%
200%	8	1.0417	1.0762	4.2%	7.6%

**Table 4:** Intermediate precision validation of the NFAT assay.

Potency Level	Run	GSD	CIGSD	GCV	CIGCV
50%	8	1.0671	1.1238	6.7%	12.4%
80%	8	1.0517	1.0947	5.2%	9.5%
100%	8	1.0737	1.1363	7.4%	13.6%
125%	8	1.0612	1.1127	6.1%	11.3%
200%	8	1.0631	1.1162	6.3%	11.6%

**Table 5:** Relative bioactivities of the rhTSH stock solutions and injections as determined using two reporter-gene assays (*n* = 3).

Samples	CRE Assay		NFAT Assay	
	Mean	RSD	Mean	RSD
DS1	111.77%	4.07%	102.09%	3.23%
DS2	98.67%	9.89%	96.21%	2.71%
DS3	95.03%	4.37%	101.56%	4.77%
DP1	98.90%	5.92%	93.58%	3.25%
DP2	94.80%	3.18%	96.14%	4.38%
DP3	99.30%	5.75%	98.45%	5.40%

## Data Availability

Data are contained within the article.
